# Comparison of four digital PCR platforms for accurate quantification of DNA copy number of a certified plasmid DNA reference material

**DOI:** 10.1038/srep13174

**Published:** 2015-08-25

**Authors:** Lianhua Dong, Ying Meng, Zhiwei Sui, Jing Wang, Liqing Wu, Boqiang Fu

**Affiliations:** 1 National Institute of Metrology, Beijing, 100029, P. R. China; 2 Hubei Institute of Measurement and Testing Technology, 430223, Wuhan, P. R. China

## Abstract

Digital polymerase chain reaction (dPCR) is a unique approach to measurement of the absolute copy number of target DNA without using external standards. However, the comparability of different dPCR platforms with respect to measurement of DNA copy number must be addressed before dPCR can be classified fundamentally as an absolute quantification technique. The comparability of four dPCR platforms with respect to accuracy and measurement uncertainty was investigated by using a certified plasmid reference material. Plasmid conformation was found to have a significant effect on droplet-based dPCR (QX100 and RainDrop) not shared with chip-based QuantStudio 12k or BioMark. The relative uncertainty of partition volume was determined to be 0.7%, 0.8%, 2.3% and 2.9% for BioMark, QX100, QuantStudio 12k and RainDrop, respectively. The measurements of the certified pNIM-001 plasmid made using the four dPCR platforms were corrected for partition volume and closely consistent with the certified value within the expended uncertainty. This demonstrated that the four dPCR platforms are of comparable effectiveness in quantifying DNA copy number. These findings provide an independent assessment of this method of determining DNA copy number when using different dPCR platforms and underline important factors that should be taken into consideration in the design of dPCR experiments.

Digital polymerase chain reaction (dPCR) is a relatively new technique. It does not require any external calibrators to measure the absolute and relative copy number of target DNA^1^,^2^. In the 1990s, the concept of digital PCR was first described^3^,^4^. Since then, dPCR has seen increasingly used for DNA quantification^5^,^6^ and as a supplement to next-generation sequencing^7^. In DNA quantification, dPCR has been utilized mostly in challenging studies involving minority targets amid complicated backgrounds such as allelic imbalance and rare mutations in cancer^8^. A typically dPCR experiment workflow has been described previously^1^,^9^. Based on Poisson statistics, the DNA copy numbers per microliter (*T*) is calculated using equation (1). Where *P* is the PCR positive partitions, *N* is the total partitions, *V*_*p*_ is the partition or droplet volume, *D* is the dilution factor combining both the factor used to dilute the DNA during PCR preparation and the factor used to further dilute the DNA with the PCR master mixture.
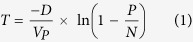


Currently, more than six commercialized digital PCR platforms are available. Some of them are microfluidic-chamber-based BioMark® dPCR from Fluidigm, micro-well chip-based QuantStudio12k flex dPCR and 3D dPCR from Life Technologies, and droplet-based ddPCR (ddPCR) QX100 and QX200 from Bio-Rad® and RainDrop from RainDance®. The microfluidic-chip-based dPCR can have up to several hundred partitions per panel. Droplet-based dPCR usually has approximately 20,000 partitioned droplets^10^ and can have up to 10,000,000 per reaction^11^. The QuantStudio 12k dPCR performs digital PCR analysis on an OpenArray® plate which contains 64 partitions per subarray and 48 subarrays in total, equating to a total of 3072 partitions per array.

The total number of analyzed partitions (*N*), the number of positive partitions/droplets (*P*) and the partition volume (*V*_*p*_) are key factors affecting the reliability of dPCR when measuring DNA concentration (see equation (1)). The total number of analyzed partitions and partition volume depends on which dPCR platform used for the measurement. For example, there are 765 partitions in each panel and each partition volume is 6 nL for a microfluidic-chip-based dPCR digital array (12 × 765) claimed by the manufacturer. The number of positive partitions/droplets in each panel/reaction is determined by the number of DNA molecule in the PCR solution. To get high accuracy and low uncertainty of dPCR measurement result, the number of positive partitions/droplets should follow in an optimal range by dispensing an optimal number of DNA targets into each panel/reaction for each dPCR system^1^,^9^. The partition/droplet volume contributes, sometimes profoundly, to the accuracy and uncertainty to dPCR measurements. The precision and evaluation of uncertainty of BioMark and QX100 dPCR measurements has been reported in previous studies^1^,^9^,^12^. However, little information is available regarding the evaluation of precision, accuracy, and uncertainty of QuantStudio 12k dPCR. Moreover, the comparability of different dPCR platforms with respect to measurement of DNA copy number must be addressed before dPCR can be classified fundamentally as an absolute quantification technique. Four representative dPCR instruments were selected for analyzing their comparability and accuracy in this study. They are chip based BioMark and QuantStudio12k flex and droplet-based QX100 and RainDrop.

The aims of this study are to evaluate the factors affecting the accuracy and measurement uncertainty of the QuantStudio 12 K Flex dPCR, and to compare the accuracy and measurement uncertainty of the four different dPCR platforms by using a certified plasmid reference material. The linearity of the response and precision over the dynamic range of the QuantStudio 12k dPCR platforms were studied. The partition (fill) volume of the QuantStudio 12k dPCR was accurately measured by gravimetric analysis. To evaluate the uncertainty and accuracy of BioMark, QX100, and RainDrop, the partition volume of these platforms was also measured. Findings collected here highlight key factors that should be taken in consideration in the use of dPCR to determine DNA copy number.

## Materials and Methods

### DNA sample and PCR assays

A purified plasmid DNA (pNIM-001) in 1 × TE_0.01_ (10 mM Tris-HCl, 0.01 mM EDTA, pH = 8.0) containing maize line NK603 event specific gene fragment (108 bp) and *zSSIIb* maize endogenous gene fragment (151 bp), constructed by National Institute of Metrology, China, was used to evaluate the dynamic range and accuracy of QuantStudio 12 K Flex digital PCR and the comparability of four dPCR platforms. The stock concentration of the plasmid was ((2.40 ± 0.14) × 10^8^) copies/μL by combining the results quantified by isotope dilution mass spectrometry (IDMS) and dPCR (the certificate of pNIM-001 in the supplementary material). Optimized Taqman probe PCR assay targeting NK603 gene fragment^13^ and reference value of this plasmid is in Table S1 and Table S2 in the supporting information. The detail information of the PCR reaction mixture condition for each dPCR instrument was listed in table S3 in the supporting information. The optimized PCR thermal profiles for BioMark contains a 10 min activation period at 95 °C followed by 50 cycles of a two steps thermal profile of 15 s at 95 °C denaturation and 60 s at 60 °C for combined annealing-extension. For droplet-based ddPCR (QX100 and RainDrop), an additional step of 10 min at 98 °C for stabilizing the droplets was added after 50 cycles. The workflow and data analysis of BioMark, QX100 and RainDrop was described^1^,^9^,^14^ in the supporting information.

### Workflow and data analysis of the QuantStudio 12 K Flex digital PCR

The digital PCR analysis was performed on a QuantStudio 12k System (Life Technologies, CA, USA) using a 48 × 64 OpenArray^®^. Each array had 48 subarrays of 64 wells each. The reaction mixture was 5 μL in volume and comprised 2.5 μL of 2 × Taqman® OpenArray® Master Mix (Life Technologies), 0.25 μL of 20× primers and probe mixture, and 1 μL of template DNA. No template control was prepared by adding same amount of 1 × TE_0.1_ in place of DNA. The PCR reagents other than DNA template were premixed and the final reaction mix was prepared gravimetrically by combining the DNA and PCR reagents to minimize the uncertainty from pipetting. The mixed DNA and reagent was dispensed into a 384-well plate (Life Technologies). This sample plate was then covered with aluminum foil. Then the sample plate and an empty chip were placed into the OpenArray® AccuFill™ System (Life Technologies) to dispense the sample into each partition of the chip from the each well of the 384-well plate. Sample filling was performed by the hydrophilic coatings via capillary action on the OpenArray^®^ chip. The array loaded with sample was affixed with the case lid and filled slowly with immersion fluid, sealed, and loaded onto carrier of the QuantStudio^®^ to perform thermal cycling and imaging of the experiment chip. The thermal cycling consisted of a 10 min activation period at 95 °C followed by 40 cycles of a two steps thermal profile of 15 s at 95 °C denaturation and 60 s at 60 °C for combined annealing-extension. Data including the amplification curve and Cq (Quantification cycle) value were acquired by QuantStudio 12k flex software v1.1.1. Discrimination between partitions that contained target (positive) and those that did not (negative) was based on the Cq value and the quality of the amplification curve set in the DigitalSuite software v1.0 for data analysis. The calculation of the concentration copies/μL is based on equation (1). Typically 33 nL was pre-set for *V*_*P*_ in the DigitalSuite software v1.0.

To evaluate linearity and precision over the dynamic range of QuantStudio 12k dPCR, three independent gravimetric serial dilution of the plasmid sample digested with *EcoR1* were prepared and used to generate three sets of eight solutions containing an average 268, 222, 125, 73, 29, 14, 3.3, and 1.4 copies per 2.11 μL QuantStudio 12k dPCR (64 partitions per subarray and a volume of 33 nL per partition) based on the certificate of pNIM-001. Each solution was analyzed in six replicates on one chip and a total 3 chips were run.

### Determination of partition volume of the QuantStudio 12 K Flex dPCR

Initially, a coherence correlation interferometer was used to image the structure and measure the volume of the partition. After imaging the through-hole, it became very difficult to determine volume because the through-hole was wider at both ends than in the middle rather than a perfect cylinder. Alternatively, measuring the weight of liquid capable of filling the through hole was used to determine the partition volume. In this way, the partition volume of the OpenArray^®^ was indirectly measured by determining the difference between the weights of an empty chip and chip loaded with PCR solution using a six-figure balance (XP56, Mettler Toledo). However, it is very difficult to balance a chip after filling it with PCR solution because of volatilization. Finally, the difference in weight between the unloaded chip and filled chip covered with the case lid (to eliminate any difference attributable to volatilization) was measured. After loading DNA and PCR reagents, the chips were covered with the chip case lid and weighed on the balance. To check the exact number of wells filled with PCR solution, the chips were loaded onto the carrier to image with the software. For measuring intra-chip variation, two chips were loaded with the first two rows of 24 subarrays, two chips were loaded with the last two rows of 24 subarrays, and two chips were loaded with all 48 subarrays. Six chips were measured in total. The measuring workflow refers Figure S1 in the supporting information. To address inter-chip variation, one row was filled each time and four measurements were taken per chip. The fill volume can be calculated using equation (2).
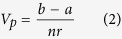


Here, *b* is the mass of the chip loaded with sample and covered with the lid; *a* is the mass of the unloaded chip with the lid; *r* is the density of PCR master mix; and *n* is the number of partitions loaded with DNA sample.

### Measurement of droplet volume of QX100 and RainDrop digital PCR

The droplets were loaded into microscopy chambers and covered with covers lips and images were captured with a MicroPublisher RTV CCD camera. The images of the droplets were scanned by an Olympus microscope with a 5× field lens for QX100 and both a 10× field lens and a 20× field lens for RainDrop. The images were analyzed using image analysis software (OmniMet, Buehler). The edge of the droplet was defined by setting up the threshold in the OmniMet software. Circles were delineated and filled based on the threshold. The equivalent circular area in (pixels)^2^ and diameter in pixels were calculated only for filled circles with sphericity >0.75. The equivalent circular diameter was converted to units of length. Finally, the equivalent spherical volume of each sphere was calculated based on the equivalent circular diameter. A total of 685 droplets from 4 different channels were measured for QX100. For RainDrop, 986 droplets from 8 channels located in three different cartridges were measured using the 20× field lens and 2068 droplets from 4 channels of two different cartridges were measured using the 10× field lens.

### Enzymatic digestion of the plasmid

The pNIM-001 plasmid was linearized by *EcoR*1 (Takara) which does not target any sequence in the PCR amplicon before analyzing its quantity using four digital PCR platforms. Enzymatic digestion of mixture comprised of 2 μL 10× buffer, 1 μL *EcoR1* (15 U/μL) restriction enzyme, 10 μL plasmid pNIM-001 DNA, and 7 μL ddH_2_O. No template control (NTC) was prepared by adding 10 μL 1 × TE_0.1_ (10 mM Tris-HCl, 0.1 mM EDTA, pH = 8.0) instead of the DNA solution, and no enzyme control (NEC) was made by pipetting 1 μL 1 × TE_0.1_ in place of the enzyme when preparing the enzymatic master mix. The enzymatic reaction lasted for 1 h at 37 °C and inactivated for 15 min at 65 °C. After the enzymatic reaction, the DNA was diluted to suitable concentrations for analysis on the different digital PCR platforms. Same plasmid DNA solutions with different concentration were mixed with prime probe and PCR master mix to be analyzed on four digital PCR platforms.

### Comparison of accuracy and measurement uncertainty of different dPCR platforms

To compare the accuracy in quantification measurement by four different digital PCR platforms, the certified plasmid DNA pNIM-001 was digested by *EcoR1* and gravimetrically diluted according to its concentration to give predictλvalues (mean copies per partition) between 1.0 and 1.6 to obtain the optimal precision for each dPCR instrument^15^. The dilution factor and required information^16^ for each dPCR instrument was listed in table S3 (Supporting Information). Quantification experiments were performed 5 replicates on three separate vials per dPCR platform. Two NTCs were prepared by adding same amount of 1 × TE_0.1_ in place of the DNA solution to exclude the contamination one each dPCR instrument.

The number of analyzed partitions, *N*, was set to 64 for QuantStudio 12k. However, for BioMark using a 12.765 chip, *N* was set to 765. For ddPCR, the value depended on the number of accepted droplets, which was between 11,000 and 20,000 for QX100 and between 1,500,000 and 3,000,000 for RainDrop. For more detail information of the analyzed partitions and positive partitions, please refer table S3, S5-S8 in the supporting information. *V*_*P*_, the partition or droplet volume of each dPCR platform was defined in this study. The uncertainty for *T*, which was related to the volume of the partition/droplet (*V*_*P*_), copy number per panel (*M*), and dilution factor (*D*), was calculated using equation (2) and (3) in earlier report^12^. The relative standard uncertainty of the partition volume 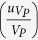, was determined using gravimetric analysis combining balance calibration, density measurement, and relative standard deviation of the gravimetrical measurement of partition volume of QuantStudio 12k dPCR. For ddPCR, the uncertainty of droplet volume was determined through analysis of an individual droplet image. Factors such as the tightness of the elliptical fit, microscope calibration, and focus were all taken into account in the estimation of uncertainty. The expanded uncertainty was calculated by multiplying the combined standard uncertainty by a coverage factor (*k* = 2). This provided a level of confidence of 95%.

### Data Analysis

The estimated copy number data generated from each dPCR platform was calculated by using equation (1). Grubbs tests were firstly performed to check if there are outliers and then T test was used to check the significance between platforms.

## Results and Discussion

### Evaluation of QuantStudio 12k digital PCR

#### Linearity and precision of QuantStudio

**Figure 1:**
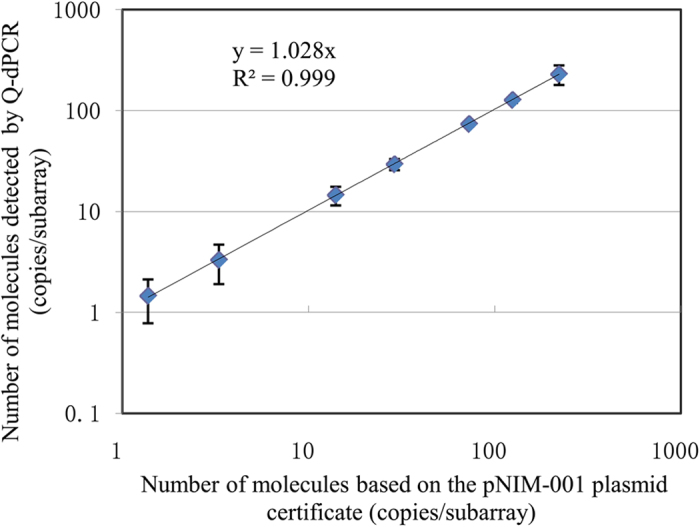
Linearity of QuantStudio digital PCR response. Symbols denote the estimated number of molecules per subarray (vertical bars denote the standard deviation from six replicate subarrays) using the NK603 event specific assay of pNIM-001 plasmid DNA.

We prepared a nominal number ranging from approximately 1.4 to 268 copies per subarray of QuantStudio 12k calculated assuming a total volume per subarray of 2.11 μL (manufacturer’s specifications) and a certified value of 2.4 × 10^8^ copies/μL. A linear relationship (r^2^ = 0.999; Fig. 1) was observed between the estimated number of molecules per subarray which ranged from 1.4 ± 0.6 to 222 ± 50 nominal copies. The estimated number of molecules was very close to the assigned value. The theoretical dynamic range in digital PCR was mainly determined by the number of partitions analyzed. The linearity range of QuantStudio 12k determined by the dynamic range of QuantStudio 12k OpenArray^®^ chip was not too wide because to the OpenArray^®^ used in this study has only 64 partitions per technical replicate.

**Figure 2:**
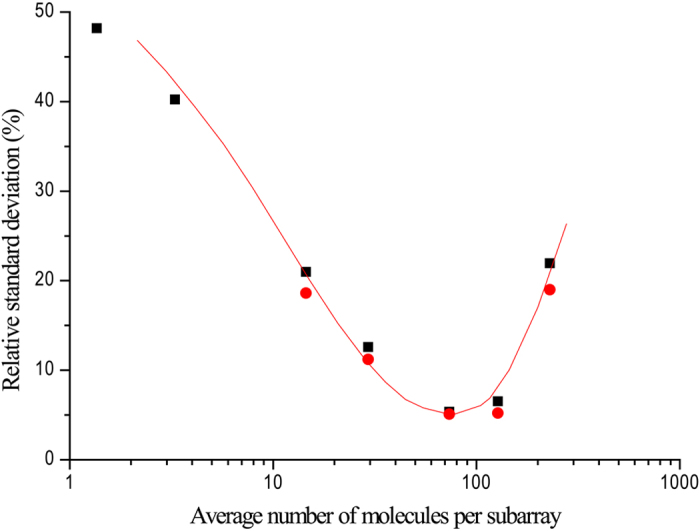
Relative standard deviation of QuantStudio 12k digital PCR response. Average number of molecules per subarray (M) as indicated on 6 (square) and 24 (dot) replicates subarrays using the NK603 event specific assay of pNIM-001 plasmid DNA.

However, the precision of measurements performed using QuantStudio 12k was not constant across the linearity range, as previously described for BioMark and QX100^1^,^9^,^12^. Precision is usually expressed numerically in measures of imprecision, such as relative standard deviation (RSD). As the concentration of the DNA sample decreases, the number of DNA targets in the replicate varies more profoundly. This is directly reflected in the RSD of the DNA copy number per subarray: the DNA copy numbers were lower than 30 and the RSDs for these were larger than 10%. As the number of copies per subarray increased, the RSD of the analyzed results decreased. The QuantStudio 12k achieved highest precision (RSD < 6%) when the copies of the template per subarray was between 52 and 104. The data was generated from six replicate subarrays and it was confirmed by analysis of 24 replicate subarrays containing 51 to 104 template molecules per subarray (Fig. 2). When the number of copies per subarray was outside of this range, the RSD increased due to either the stochastic effect or to near-saturation of the dPCR chip.

It has been reported that the linearity range of BioMark^1^ and QX100^12^ was more than two and four orders of magnitude, respectively. The linearity of QuantStudio 12k is relative narrow compared with the BioMark and QX100. However, it is more proper to measure trace DNA due to its high precision when the copies of the template per subarray was between 52 and 104. All other three dPCR platforms cannot achieve such a high precision when measuring such low concentration of target DNA. This is advantageous of QuantStudio 12k among the other three dPCR platforms.

#### Measurement and uncertainty assessment of partition volume of QuantStudio

The concentration of target DNA in the solution was here estimated using binomial approximation based on the number of positive partitions and the total number of partitions analyzed^17^. To quantify DNA concentration using dPCR, two key factors were found to influence the reliability of dPCR measurements, the number of partitions analyzed and the number of target DNA molecules in the PCR assay. However, the partition volume (*V*_*P*_) needs to be considered when measuring absolute DNA concentration by dPCR. This is because concentration is derived by dividing the estimated copy number by the partition volume.

The partition volume of QuantStudio 12k was successfully analyzed by measuring the fill-volume of the through-holes of the OpenArray® chip by gravimetric analysis. First, Coherence Correlation Interferometry (Talysurf CCI-Lite Non-contact 3D Profiler, CCI) imaging was performed on one digital array loaded with PCR solution to determine the partition volume. Each subarray comprises 8 rows and 8 columns making up the 64 partitions. The partitions are approximately cylinder and the approximate volume of the cylinder was (34.18 ± 0.34) nL measured using the CCI (Figure S2 in the supporting information). However, according to the manufacturer, the partition is not perfect cylinder, being wider at both ends than in the middle (Life Technologies, personal communication), somewhat like intersecting cones or hemispheres. This adds to the dry partition volume. The actual fill volume differs from this dry volume due to the meniscus of the liquid after loading. The meniscus is formed by the patterned hydrophilic and hydrophobic coatings that were also used to ensure filling of the through-holes. When filled with liquid, the aqueous solution forms a positive meniscus—the fluid bulges out at either end of the hole. This further increases the volume. Finally, the fill volume of the through-holes was measured by gravimetric analysis. The determined average fill volumes with an expanded uncertainty were (32.7 ± 1.2) nL inter-chip and (32.8 ± 1.5) nL intra-chip. All partition volume values were found to be equivalent to the volume claimed by the manufacturer considering the measurement uncertainty. The expanded relative uncertainty for the fill volume at 95% confidence was 2.3%, which includes the uncertainty from precision factor of the gravimetric measurement and uncertainty from the calibration of the balance used (Figure S3 in the supporting information).

### Comparability of four different digital PCR platforms

#### Effects of plasmid conformation on quantification by four dPCRs

The plasmid conformation has a significant effect on droplet based QX100 according to an earlier report^12^. Thus, the impact of plasmid conformation on QuantStudio 12k and other three dPCR platforms was investigated by using a certified plasmid reference material. The measured concentrations of linearized and unlinearized pNIM-001 on four dPCR platforms were compared in Table 1. The copy number concentration of linearized plasmid was not significantly different from that of unlinearized plasmid on QuantStudio 12k (*P* = 0.17) and BioMark (*P* = 0.32). However, the copy number concentrations of linearized plasmid were far higher than those of unlinearized plasmid as determined on QX100 (*P* = 6.25 × 10^−6^) and RainDrop platform (*P* = 1.08 × 10^−4^). Results indicate that the pNIM-001 plasmid conformation has a clear effect on target DNA quantification by droplet format ddPCR and not by QuantStudio 12k or BioMark. This was confirmed by another PCR assay targeting the *zSSIIb* gene fragment in the same plasmid (data not shown). Except QX100, same PCR master mixs were used for QuantStudio 12k, BioMark and RainDrop, which excludes the possibility of underestimation of plasmid copy number on RainDrop caused by different PCR regent. The underestimation of copy number concentration of unlinearized plasmid on ddPCR may have been caused by unsuccessful amplification of target DNA molecules.

**Figure 3:**
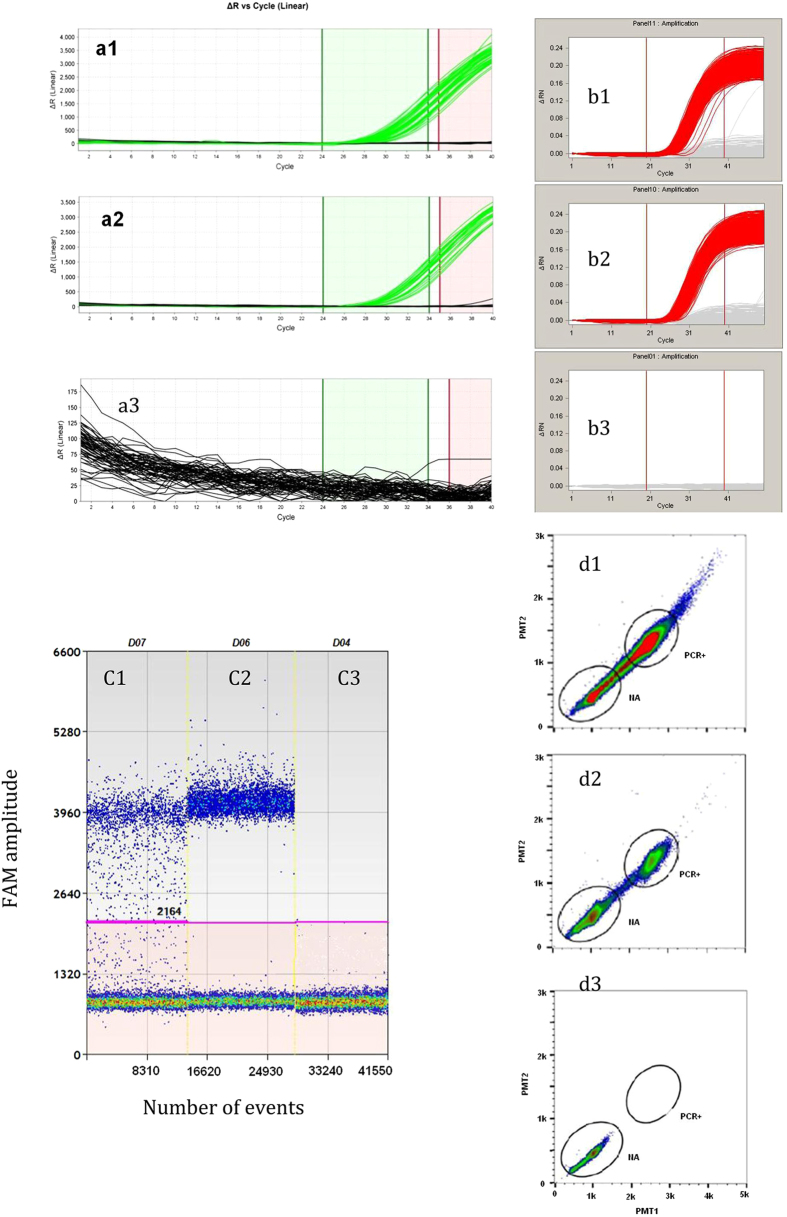
Amplification with unlinearized plasmid (a1, b1, c1, and d1), linearized plasmid (a2, b2, c2, and d2) and not template control (a3, b3, c3 and d3) ccompared by targeting an NK603-event specific assay on QuantStudio 12k, BioMark, QX100, and RainDrop digital PCR.

The Cq values were between 28.51 and 30.25 for most positive partitions on QuantStudio 12k (Fig. 3a). This relatively concentrated distribution of Cq values for most partitions on QuantStudio 12k indicates that most molecules had similar amplification efficiency. Thus, there was no significant difference (*P* = 0.17) in copy number measured between unlinearized and linearized plasmid (Fig. 3a1,a2). For BioMark, like QuantStudio 12k, the Cq value for unlinearized plasmid was very similar to that of linearized plasmid (Fig. 3b1,b2) and no spread in Cq values for unlinearized plasmid was observed. This observation differs from that of previous reports, in which single-molecule amplification from some unlinearized plasmid molecules was clearly delayed^1^. This was deduced to be possibly plasmid-dependent or different PCR master mix used. The one-dimensional scatter plots for selected wells with unlinearized or linearized plasmid on QX100 and RainDrop are listed in Fig. 3c,d. A smear between the positive and the negative clusters was observed for the unlinearized plasmid on both ddPCR platforms. This indicates a delay in amplification in these smeared droplets. However, the smear was greatly reduced by linearizing the plasmid, suggesting that plasmid conformation is a significant factor for ddPCR quantification. This confirmed that underestimation of unlinearized plasmid copy number is partially caused by delay in the amplification of ddPCR.

For the real time QuantStudio 12k and BioMark dPCR, the advantage is amplification curve is available, thus the target Cq range for positive partitions can be set by the operator. However, if the target Cq range for positive partitions excludes those with higher Cq values (this would be done on the assumption that these higher Cq values represent non-specific amplification), then the true number of molecules in a subarray or panel for which all partitions have Cq values greater than the target Cq range may be underestimated because they will be not be recognized as positive partitions^1^. For this reason another parameter, the quality of the amplification curve, should be taken into account when setting the target Cq range. The discrimination of positive and negative is a comprehensive evaluation of both the Cq value and the quality of the amplification curve for real-time digital PCR. However, the amplification curve is unavailable for ddPCR. This is because ddPCR is end-point reading. It therefore cannot determine the number of positive droplets based on Cq values. Though the positive and negative partitions can be discriminated by manually setting the threshold, it would be not easy to set if a smear occurred. This makes amplification efficiency much more important for ddPCR quantification.

#### Evaluation of measurement uncertainty of droplet/partition volume of four dPCRs

**Figure 4:**
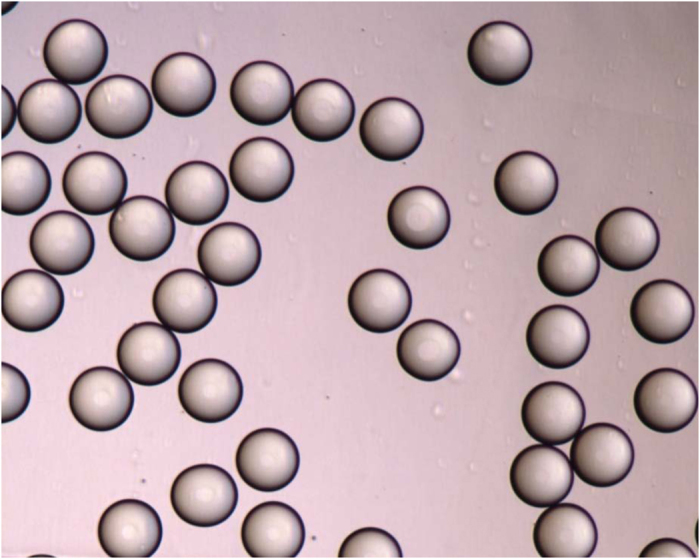
Droplets generated from QX100 digital PCR as scanned by Olympus microscope with a 5× field lens.

The droplet volume of QX100, which is a major source of uncertainty, has been evaluated previously^9^,^12^. The uncertainty of QX100 droplet volume measurement was found to be 1% in a previous report and the measurement was improved by using high-resolution microscopy with a wide-field CCD^12^. This allowed more droplets to be imaged per view, and 4 times more droplets were measured than in an earlier study (Fig. 4). The average volume was calculated to be 0.837 nL based on 685 droplets measured from four channels across three cartridges (Table S4) and the relative uncertainty of droplet volume was 0.8%, including the variability of droplet volume, microscopy calibration, and effects of the focal plane (Figure S3 in the supporting information).

**Figure 5:**
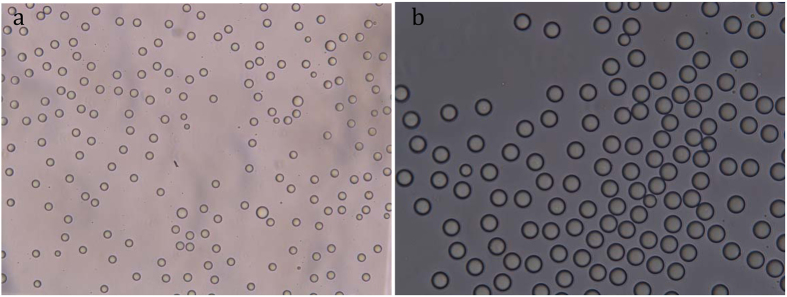
Droplets generated from RainDrop digital PCR as scanned by Olympus microscope with a (**a**) 10× and (**b**) 20× field lens.

Droplet volume was measured, for the first time, for RainDrop. The average droplet volume is claimed to be 5 pL by the manufacturer, so the droplets on RainDrop were here assumed to be about 160 times smaller than those generated on the QX100. Here, 10× and 20× field lenses were used to image the smaller droplets (Fig. 5). The average sizes of the droplets measured using the 10× field lens and 20× field lens were 4.39 pL and 4.47 pL, respectively. As the diffraction phenomena become more pronounced under 20× field lens it becomes difficult to distinguish the interface of the sphere. For this reason, 4.39 pL was measured using a 10× field lens and results served as the average size of droplet for DNA copy number concentration calculation. The relative uncertainty was calculated to be 2.9%, including the variability of droplet volume, microscopy calibration, and effects of focal plane.

For the BioMark platform, the partition volume of 12 × 765 digital array was measured using a Raman microscope (InVia, Renishaw). One partition per panel was analyzed and there were a total 12 partitions per array. For each panel, one partition at the intersection of row 9 and column 10 was analyzed. The lateral dimensions in the x-y plane were determined from images focused on the top of the partitions (Figure SR1a in the supporting information). For measurement along the z-axis, the chip was cut along with x axis and the x-z cross-section was imaged and measured. The average volume with uncertainty was determined to be (6.70 ± 0.05) nL, which is relatively larger than in a previous report^1^. This discrepancy could be attributed to their limited resolution of the z-axis measurement. In previous report, the measurement of z-axis was achieved by measuring the distance between top focus and bottom focus, which could generate large variation on z-axis measurement. In our study, the z-axis measurement was treated as a lateral dimension measurement, which would be much more accurate. The relative uncertainty was 0.7%, including the variability of partition volume, microscopy calibration, and effects of the focal plane.

In summary, the relative uncertainty of the partition/droplet volume (type B component) from small to large was as follows: BioMark (0.7%), QX100 (0.8%), QuantStudio 12k (2.3%), and RainDrop (2.9%). The proportion of uncertainty from precision data and the volume of the partition, and other sources of uncertainty for all four dPCR platforms were investigated. Results are shown in Figure S3 of the supporting information. The relative amount of uncertainty in the precision data was found to be 93.95%, 87.75%, 43.94%, and 20.91% for BioMark, QX100, QuantStudio 12k, and RainDrop, respectively. The uncertainty on partition or droplet volume for BioMark, QX100, QuantStudio 12k, and RainDrop was 4.92%, 8.74%, 55.44%, and 78.16%, respectively. The partition/droplet volume for QuantStudio 12k and RainDrop was successfully measured for the first time. This made it possible to evaluate the uncertainty in the partition volume for these two digital PCR platforms. Uncertainty was greatly decreased by reducing the measurement uncertainty on partition volume for BioMark. As described in an earlier paper^1^, the z-axis of the partition is the major contributor to the uncertainty of the BioMark partition volume, and the uncertainty in the z-axis dimension is attributable both to the limited resolution of the z-axis measurement and the variation in the z-axis dimension across panels. The limited resolution of the z-axis measurement was improved by an alternative measurement: z-axis measurement was treated as a lateral dimension measurement in that the x-z cross sectional was imaged and measured under the microscope by cutting the chip along the x-axis.

#### Comparison of accuracy in DNA concentration measurements using four dPCRs

**Figure 6:**
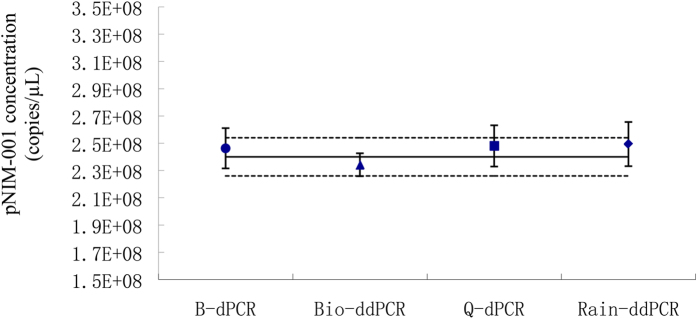
Stock concentrations with the expanded uncertainty of the certified plasmid DNA measured by BioMark digital PCR (B-dPCR), BioRad QX100 droplet digital PCR (Bio-ddPCR), QuantStudio 12 K digital PCR (Q-dPCR), and RainDrop droplet digital PCR (Rain-ddPCR). All the dPCR measurement values were corrected with its individual partition volume. The certified concentration for the plasmid DNA stock (black line) with the expanded uncertainty (dash lines) was calculated by combining isotope dilution mass spectrometry and B-dPCR with correction of partition volume.

The measured DNA concentration and its uncertainty for the pNIM-001 by four dPCR platforms are shown in Table 2 and Fig. 6. The measured result by four dPCR platforms does overlap within their expanded uncertainty. Accuracy and precision are critical to evaluating the performance of an analytical method. Conventionally, the accuracy of a measurement is its closeness to a measured quantity value and a true quantity value of a measurand^18^. In practice, no true value can be fully acquired, and the accuracy of measurement must be understood as the degree of similarity between measured quantity values that are being attributed to the measurand. For dPCR measurement, typically, the accuracy is the closeness of a measured DNA quantity and a true quantity value of DNA concentration. All four dPCR systems provide a measurement of the absolute copy number concentration. For the measurement of certified plasmid DNA by dPCR, the accuracy can be assigned to be the closeness between the result obtained from each independent dPCR platform and the certified value.

The certified plasmid DNA concentration is (2.40 ± 0.14) × 10^8^ copies/μL (*k* = 2) characterized by isotope dilution mass spectrometry (IDMS) and BioMark, as stated by the National Institute of Metrology, China. For QX100 measurement, the droplet volume was 0.837 nL, measured with more than 685 droplets, which is slightly smaller than a previous measurement of 0.846 nL^12^. In this way, the new value of the droplet volume was used for ddPCR calculation. For RainDrop, 4.39 pL of the droplet volume was used to calculate the final concentration. With the partition/droplet volume correction for each dPCR platform, the measurement of plasmid concentration by BioMark, QX100, QuantStudio 12k, and RainDrop was found to be closely with the certified value within their uncertainty. They were 103%, 97.5%, 103%, and 104% of the certified value, respectively. However, without the correction of the partition/droplet volume, the results of measurement were not comparable. This was especially true of BioMark and QX100 (Figure S4 in the supporting information). The measured value with a small uncertainty obtained from QX100 was the closest to the certified value. Full understanding of the measurement bias and uncertainty of each digital PCR platform may help users render the measurement results more accurate and comparable across different platforms.

## Conclusions

The findings in this study provide a range of information on the utility of dPCR for quantifying absolute DNA copy numbers. Plasmid conformation had a significant effect on droplet based digital PCR (ddPCR) not observed with micro-well chip-based QuantStudio 12 K and chip-based BioMark. The user can detect DNA targets as scarce as a single copy/subarray using QuantStudio 12k instruments. Droplet/partition volume, a major factor affecting the accuracy and uncertainty of dPCR measurement, was fully evaluated for all four dPCR platforms. The measurements of the certified plasmid DNA obtained from each dPCR platform with correction of droplet/partition volume overlapped well within the expanded uncertainty.

## Additional Information

**How to cite this article**: Dong, L. *et al*. Comparison of four digital PCR platforms for accurate quantification of DNA copy number of a certified plasmid DNA reference material. *Sci. Rep*. **5**, 13174; doi: 10.1038/srep13174 (2015).

## Supplementary Material

Supplementary Information

## Figures and Tables

**Table 1:** Quantitation result of unlinearized plasmid and linearized plasmid analyzed on QuantStudio 12 K flex and BioMark, QX100 and RainDrop.

Digital PCR platform	Linearized plasmid(copies/μL) (*n* = 3)	Unlinearized plasmid(copies/μL) (*n* = 3)	*P*
QuantStudio 12k	(2.47 ± 0.07) × 10^8^	(2.37 ± 0.07) × 10^8^	0.17
BioMark	(2.46 ± 0.14) × 10^8^	(2.33 ± 0.14) × 10^8^	0.32
QX100	(2.34 ± 0.06) × 10^8^	(1.08 ± 0.04) × 10^8^	6.25E-6
RainDrop	(2.49 ± 0.11) × 10^8^	(1.13 ± 0.11) × 10^8^	1.08E-4

**Table 2:** Copy number concentration and its uncertainty of pNIM-001 plasmid reference material analyzed using four digital PCR platforms.

dPCR platform	BioMark	QX100	QuantStudio12k	RainDrop
Partition number	765	13800 ± 464*	64	1695000 ± 24862*
λ (Mean copies/partition)	1.56	1.54	1.54	1.51
Measured pNIM-001 plasmid concentration	2.46E + 08	2.34E + 08	2.48E + 08	2.49E + 08
*n* (number of observation)	15	15	15	15
Relative standard uncertainty of all precision factors 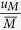 (%) (*M*, copies per panel)	2.9	1.6	2	1.5
Relative standard uncertainty of dilution factor 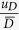 (%) (*D*, dilution factor)	0.1	0.1	0.2	0.1
Relative standard uncertainty of a single droplet/partition volume 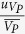 (%) (*V*_*p*_, partition volume)	0.7	0.8	2.3	2.9
Relative combined uncertainty *u* (%)	3.0	1.8	3.1	3.3
Relative expanded uncertainty *U*_*rel*_ (*k* = 2) (%)	6.0	3.6	6.1	6.5

^*^Mean with standard deviation of 15 replicates.
