# Supplementary material for: Comparison of four digital PCR platforms for accurate quantification of DNA copy number of a certified plasmid DNA reference material
Source: Sci Rep. 2015 Aug 25;5:13174. doi: 10.1038/srep13174 (PMC4548226; doi:10.1038/srep13174)
Supplement: Supplementary Information [file srep13174-s1.doc]

**Comparison of four digital PCR platforms for accurate quantification of DNA copy number of a certified plasmid DNA reference material**

Lianhua Dong^1*^, Ying Meng^2^, Sui Zhiwei^1^, Jing Wang^1^, Liqing Wu^1^, Boqiang Fu^1^

1. National Institute of Metrology, Beijing, 100013, P.R.China,
2. Hubei Institute of Measurement and Testing Technology, 430223, Wuhan, P.R.China

* Corresponding author: [donglh@nim.ac.cn](mailto:donglh@nim.ac.cn); National Institute of Metrology, Beijing, 100013, P.R.China.

**Supporting information**

**1. Workflow and data analysis of QX100, RainDrop and BioMark digital PCR**

**QX100 dPCR instrumentation and data analysis.** The QX100 dPCR workflow and data analysis were performed as described by Pinheiro [9]. Reaction mixtures of 20 µL volume comprising 10 µL of 2× ddPCR Master Mix (Bio-Rad), 1 µL of 20×primers and probe mixture (Table S3 in the Supporting Information) and 5 µL of 1×TE_0.1_ (10 mM Tris-HCl, 0.1 mM EDTA, pH=8.0). The PCR regents were premixed in a pre-PCR room to limit the risk of reagent contamination and were gravimetrically mixed with suitable concentration of DNA (ratio of 4: 1) which was gravimetrically diluted (Table S3). No template control (NTC) was prepared by adding same amount of 1×TE_0.1_ in place of the DNA solution. Each 20 µL volume of reaction mixture was transferred into a separate well of a eight channel droplet generator cartridge and 60 µL of droplet generation oil was loaded into each of a corresponding oil well. After covering with a cushion, the cartridge was transferred into droplet generator (Bio-Rad) to create water-in-oil emulsion. About 40 µL of water-in-oil emulsion from each well was transferred into a 96-well plate (Eppendorf) and then heat sealed with foil, and amplified in a thermal cycler (9700, Applied Biosystems) . The thermal cycling condition comprised of a 10 min activation period at 95°C followed by 50 cycles of a two steps thermal profile of 15 s at 95°C denaturation and 60 s at 60°C for combined annealing-extension, then a step of 10 min at 98°C for stabilizing the droplets was added after 50 cycles. After thermal cycling, the plate was transferred to a droplet reader (Bio-Rad) to read the droplets. The data was analyzed by data analysis software of QuantaSoft (version 1.2.10.0, Bio-Rad).

**RainDrop dPCR instrumentation and data analysis.** The experiment performed on RainDrop dPCR (RainDance Technologies) was conducted at Peking University. Reaction mixtures of 25 µL volume comprising 12.5 µL of 2×Taqman® OpenArray® Master Mix (Life Technologies), 1.25 µL of 20×primers and probe mixture (Table S3 in the Supporting Information), 2.5 µL of stabilizer (RainDance Technologies) and 3.75 µL of 1×TE_0.1_. The above PCR regents were premixed and then were gravimetrically mixed with proper diluted DNA solution (ratio of 4: 1) (Table S3). For the preparation of NTC, the PCR regent mixture was mixed with same amount of 1×TE_0.1_ instead of the DNA solution. Each 25 µL volume of sample was pipette into each well of a 8-channel source chip (RainDance Technologies) and loaded into the Source instrument (RainDance Technologies) following operating guidelines, to generate water-in-oil droplets. The RainDrop® Source instrument uses real-time closed-loop image control to ensure uniformity of droplet creation both within and across runs. Each 25 µL sample was emulsiﬁed into picoliter-scale droplets, partitioning single molecule of DNA into over 1 million droplets. Following emulsion generation on the RainDrop® Source instrument, the samples were thermal cycled on a conventional PCR thermal cycler (Bio-Rad). The PCR thermal profile was same as that for QX100. The thermal cycled sample was loaded onto the Sense instrument (RainDance Technologies) to read the fluorescence of the droplets. The RainDrop® Sense instrument uses a 488 nm laser to read the FAM fluorescence intensity of each droplet. After evaluating all samples using the RainDrop® digital PCR System, data from cluster plots were spectrally-compensated and analyzed using the RainDrop Analyst data analysis software.

**BioMark dPCR instrumentation and data analysis.** The workflow of BioMark dPCR platform has been described in an earlier report [1]. All PCR reactions were prepared to 10 µL volumes containing 5 µL of 2×Taqman® OpenArray® Master Mix (Life Technologies), 0.5 µL of 20×primers and probe mixture (Table S3), 0.5 µL of sample loading regent (Fluidigm) and 2 µL of 1×TE_0.1_. The premixed PCR regent was gravimetrically mixed with proper diluted DNA solution (Table S3). Each 10 µL volume of reaction mix was aliquoted into each sample inlet on the 12×765 digital chip with approximately 5 µL of the sample mixture distributed throughout the partitions within each panel using an Integrated Fluidic Circuit (IFC) Controller (Fluidigm). No Template Controls (NTC) containing 1× TE_0.1_ buffer in place of DNA was setup in two on each chip. Digital array thermal cycling condition was comprising of a 10 min activation period at 95°C followed by 50 cycles of a two steps thermal profile of 15 s at 95°C denaturation and 60 s at 60°C for combined annealing-extension. The data was analyzed by Fluidigm digital PCR analysis software (Fluidigm) using a manually set quality threshold of 0.02 and target Ct range of 15−35.

Table S1 Sequence of the primer and probe for NK603 event specific TaqMan probe digital PCR assay used for pNIM-001 plasmid DNA

| Primer/probe | Sequence (5’-3’) | Concentration(nM) | Amplicon |
| --- | --- | --- | --- |
| Forward primer NK603-F | ATGAATGACCTCGAGTAAGCTTGTTAA | 250 nM | 108bp |
| Reverse primer NK603-R | AAGAGATAACAGGATCCACTCAAACACT | 250 nM |
| Probe NK603-P | FAM-TGGTACCACGCGACAGACTTCCACTC-BHQ1 | 125 nM |

Table S2. The concentration of the pNIM-001 plasmid certified reference material

| Number | Certified value  （copies/μL） | Expanded uncertainty（*k*=2）（copies/μL） |
| --- | --- | --- |
| GBW 10086 | 2.40×10^8^ | 0.14×10^8^ |

Table S3. The detail PCR information of quantifying pNIM-001 plasmid by four digital PCR platforms.

| dPCR platform | | QuantStudio12k | BioMark | QX100 | RainDrop |
| --- | --- | --- | --- | --- | --- |
| DF (Dilution with the enzymatic mixture) | Vial-1 | 2.01 | 2.01 | 2.01 | 2.01 |
|  | Vial-2 | 2.04 | 2.04 | 2.04 | 2.04 |
|  | Vial-3 | 2.03 | 2.03 | 2.03 | 2.03 |
| DF (Dilution with 1× TE_0.1_) | Vial-1 | 526859.58 | 107761.09 | 12758.63 | 72.47 |
|  | Vial-2 | 518071.30 | 104889.20 | 12544.97 | 71.07 |
|  | Vial-3 | 522719.00 | 104981.50 | 12632.83 | 71.27 |
| DF (Dilution with PCR regent) | Vial-1 | 4.98 | 4.89 | 4.95 | 4.95 |
|  | Vial-2 | 4.99 | 4.95 | 4.96 | 4.98 |
|  | Vial-3 | 4.97 | 4.97 | 4.95 | 4.97 |
| 2×Mastermix Brand/volume (μL) | | OA/2.5 | OA/5 | BioRad/10 | OA/12.5 |
| DNA template (μL) | | 1 | 2 | 4 | 5 |
| Reaction volume (μL) | | 5 | 10 | 20 | 25 |
| 20×primer/probe(μL) | | 0.25 | 0.5 | 1 | 1.25 |
| Partition number | | 64 | 765 | 13800±464^a^ | 1695000±24862^a^ |
| Individual partition volume (nL) | | 32.80 | 6.70 | 0.837 | 0.00439 |
| Total volume of the partitions measured (μL) | | 2.10 | 5.12 | 11.55 ^b^ | 7.44 ^b^ |

^a^, mean accepted droplet number of 15 replicates;

^b^, average partition volume of accepted droplet number of 15 replicates;

DF, dilution factor; OA, 2×Taqman® OpenArray® Master Mix from Life Technologies; BioRad, BioRad master mix for probe assay from Bio-Rad.

Table S4. Droplet volume of BioRad QX100 droplet digital PCR

| Number | Volume (nL) | | | |
| --- | --- | --- | --- | --- |
|  | Channel 5 | Channel 3 | Channel 4 | Channel 2 |
| 1 | 0.7874 | 0.6764 | 0.7524 | 0.7736 |
| 2 | 0.8169 | 0.6988 | 0.7584 | 0.7779 |
| 3 | 0.8206 | 0.8304 | 0.7900 | 0.7837 |
| 4 | 0.8508 | 0.8480 | 0.8663 | 0.7993 |
| 5 | 0.8664 | 0.8586 | 0.8744 | 0.8141 |
| 6 | 0.8687 | 0.8587 | 0.7592 | 0.8550 |
| 7 | 0.7964 | 0.8711 | 0.7836 | 0.8607 |
| 8 | 0.8305 | 0.8739 | 0.8153 | 0.8740 |
| 9 | 0.8325 | 0.8804 | 0.8156 | 0.9190 |
| 10 | 0.8578 | 0.8813 | 0.8251 | 0.7682 |
| 11 | 0.8777 | 0.8846 | 0.8329 | 0.7721 |
| 12 | 0.7107 | 0.8892 | 0.8690 | 0.7726 |
| 13 | 0.7431 | 0.8946 | 0.7666 | 0.7735 |
| 14 | 0.7732 | 0.9093 | 0.7819 | 0.7821 |
| 15 | 0.7909 | 0.9155 | 0.8157 | 0.7972 |
| 16 | 0.7934 | 0.9160 | 0.8492 | 0.8119 |
| 17 | 0.8088 | 0.9232 | 0.8627 | 0.8515 |
| 18 | 0.8127 | 0.6783 | 0.7989 | 0.8580 |
| 19 | 0.8413 | 0.6994 | 0.8066 | 0.8690 |
| 20 | 0.8543 | 0.8281 | 0.8084 | 0.8697 |
| 21 | 0.8580 | 0.8369 | 0.8114 | 0.8736 |
| 22 | 0.8602 | 0.8468 | 0.8138 | 0.8753 |
| 23 | 0.6964 | 0.8548 | 0.8138 | 0.9153 |
| 24 | 0.7196 | 0.8567 | 0.8165 | 0.7720 |
| 25 | 0.7230 | 0.8572 | 0.8166 | 0.7908 |
| 26 | 0.8029 | 0.8677 | 0.8191 | 0.8019 |
| 27 | 0.8053 | 0.8695 | 0.8219 | 0.8167 |
| 28 | 0.8088 | 0.8751 | 0.8230 | 0.8424 |
| 29 | 0.8171 | 0.8829 | 0.8364 | 0.8533 |
| 30 | 0.8228 | 0.8853 | 0.8395 | 0.8613 |
| 31 | 0.8340 | 0.8901 | 0.8490 | 0.8839 |
| 32 | 0.8408 | 0.8909 | 0.8746 | 0.8896 |
| 33 | 0.8481 | 0.9155 | 0.8803 | 0.8899 |
| 34 | 0.8481 | 0.9166 | 0.8890 | 0.8903 |
| 35 | 0.8520 | 0.9247 | 0.7658 | 0.8910 |
| 36 | 0.8542 | 0.6976 | 0.7808 | 0.8932 |
| 37 | 0.8595 | 0.7663 | 0.7857 | 0.8487 |
| 38 | 0.8643 | 0.8322 | 0.7894 | 0.8644 |
| 39 | 0.8712 | 0.8532 | 0.8044 | 0.8667 |
| 40 | 0.8739 | 0.8541 | 0.8054 | 0.8684 |
| 41 | 0.9071 | 0.8592 | 0.8120 | 0.9035 |
| 42 | 0.7740 | 0.8601 | 0.8138 | 0.9825 |
| 43 | 0.7968 | 0.8805 | 0.8152 | 1.0110 |
| 44 | 0.8005 | 0.8844 | 0.8498 | 0.8100 |
| 45 | 0.8066 | 0.8952 | 0.8702 | 0.8499 |
| 46 | 0.8092 | 0.8978 | 0.8732 | 0.8517 |
| 47 | 0.8267 | 0.6922 | 0.8850 | 0.8766 |
| 48 | 0.8329 | 0.7663 | 0.7800 | 0.8780 |
| 49 | 0.8506 | 0.8069 | 0.8029 | 0.8861 |
| 50 | 0.8579 | 0.8684 | 0.8168 | 0.8906 |
| 51 | 0.8603 | 0.8798 | 0.8310 | 0.8965 |
| 52 | 0.8683 | 0.7076 | 0.8413 | 0.9016 |
| 53 | 0.8770 | 0.8376 | 0.8463 | 0.9213 |
| 54 | 0.6757 | 0.8391 | 0.8493 | 0.9249 |
| 55 | 0.7773 | 0.8532 | 0.7511 | 0.9488 |
| 56 | 0.8070 | 0.8585 | 0.7656 | 0.7778 |
| 57 | 0.8107 | 0.8659 | 0.7738 | 0.7897 |
| 58 | 0.8125 | 0.8746 | 0.7816 | 0.8248 |
| 59 | 0.8175 | 0.8805 | 0.7889 | 0.8316 |
| 60 | 0.8332 | 0.8973 | 0.8294 | 0.8513 |
| 61 | 0.8559 | 0.7067 | 0.8330 | 0.8545 |
| 62 | 0.8592 | 0.7298 | 0.8331 | 0.8569 |
| 63 | 0.8626 | 0.7688 | 0.8439 | 0.8603 |
| 64 | 0.8662 | 0.7873 | 0.8457 | 0.8629 |
| 65 | 0.8367 | 0.8214 | 0.8546 | 0.8631 |
| 66 | 0.8463 | 0.8312 | 0.7607 | 0.8851 |
| 67 | 0.8640 | 0.8378 | 0.7884 | 0.8922 |
| 68 | 0.8664 | 0.8688 | 0.7972 | 0.9019 |
| 69 | 0.8696 | 0.8815 | 0.8046 | 0.9063 |
| 70 | 0.8872 | 0.8935 | 0.8122 | 0.9156 |
| 71 | 0.9044 | 0.5723 | 0.8444 | 0.6919 |
| 72 | 0.9178 | 0.7714 | 0.8445 | 0.7763 |
| 73 | 0.8238 | 0.7843 | 0.7239 | 0.8087 |
| 74 | 0.8565 | 0.8034 | 0.7617 | 0.8133 |
| 75 | 0.8614 | 0.8477 | 0.7897 | 0.8145 |
| 76 | 0.8719 | 0.8548 | 0.7980 | 0.8386 |
| 77 | 0.8748 | 0.5708 | 0.8057 | 0.8489 |
| 78 | 0.8788 | 0.7853 | 0.8093 | 0.8513 |
| 79 | 0.8942 | 0.7962 | 0.8118 | 0.8550 |
| 80 | 0.9165 | 0.8258 | 0.8136 | 0.8635 |
| 81 | 0.6984 | 0.8297 | 0.8352 | 0.8662 |
| 82 | 0.7493 | 0.8424 | 0.8364 | 0.8739 |
| 83 | 0.7976 | 0.8477 | 0.8513 | 0.8748 |
| 84 | 0.8001 | 0.8519 | 0.8542 | 0.8790 |
| 85 | 0.8397 | 0.8551 | 0.9382 | 0.9151 |
| 86 | 0.8981 | 0.8554 | 0.7482 | 0.7701 |
| 87 | 0.8999 | 0.8563 | 0.7556 | 0.8100 |
| 88 | 0.9182 | 0.8575 | 0.7957 | 0.8124 |
| 89 | 0.9267 | 0.8622 | 0.7969 | 0.8145 |
| 90 | 0.9549 | 0.8684 | 0.8139 | 0.8147 |
| 91 | 0.9886 | 0.8694 | 0.8255 | 0.8303 |
| 92 | 0.8497 | 0.8770 | 0.8293 | 0.8471 |
| 93 | 0.9117 | 0.8779 | 0.8922 | 0.8501 |
| 94 | 0.9163 | 0.8785 | 0.9311 | 0.8627 |
| 95 |  | 0.8850 | 0.7319 | 0.8633 |
| 96 |  | 0.8943 | 0.7587 | 0.8730 |
| 97 |  | 0.9309 | 0.7709 | 0.9118 |
| 98 |  | 0.6004 | 0.7848 | 0.9780 |
| 99 |  | 0.6706 | 0.7953 | 0.7433 |
| 100 |  | 0.6828 | 0.8259 | 0.7488 |
| 101 |  | 0.7342 | 0.8447 | 0.7760 |
| 102 |  | 0.7934 | 0.8573 | 0.7968 |
| 103 |  | 0.8041 | 0.8685 | 0.8264 |
| 104 |  | 0.8171 | 0.8777 | 0.8301 |
| 105 |  | 0.8211 | 0.7180 | 0.8457 |
| 106 |  | 0.8240 | 0.7746 | 0.8474 |
| 107 |  | 0.8340 | 0.7768 | 0.8541 |
| 108 |  | 0.8385 | 0.7796 | 0.8566 |
| 109 |  | 0.8450 | 0.8030 | 0.8607 |
| 110 |  | 0.8498 | 0.8205 | 0.8615 |
| 111 |  | 0.8512 | 0.8213 | 0.8684 |
| 112 |  | 0.8526 | 0.8287 | 0.8743 |
| 113 |  | 0.8537 | 0.8368 | 0.7190 |
| 114 |  | 0.8560 | 0.8467 | 0.7215 |
| 115 |  | 0.8570 | 0.8486 | 0.7712 |
| 116 |  | 0.8613 | 0.8534 | 0.7933 |
| 117 |  | 0.8655 | 0.8586 | 0.8160 |
| 118 |  | 0.8656 | 0.8617 | 0.8418 |
| 119 |  | 0.8675 | 0.8620 | 0.8460 |
| 120 |  | 0.8675 | 0.8643 | 0.8491 |
| 121 |  | 0.8715 | 0.8662 | 0.8518 |
| 122 |  | 0.8746 | 0.8741 | 0.8602 |
| 123 |  | 0.8764 | 0.8191 | 0.8718 |
| 124 |  | 0.8779 | 0.7192 | 0.7174 |
| 125 |  | 0.8791 | 0.8030 | 0.7204 |
| 126 |  | 0.8826 | 0.8205 | 0.7691 |
| 127 |  | 0.8844 | 0.8213 | 0.7941 |
| 128 |  | 0.8881 | 0.8287 | 0.8163 |
| 129 |  | 0.8883 | 0.8368 | 0.8195 |
| 130 |  | 0.8969 |  | 0.8394 |
| 131 |  | 0.9003 |  | 0.8718 |
| 132 |  | 0.9017 |  | 0.7054 |
| 133 |  | 0.9309 |  | 0.7846 |
| 134 |  | 0.8287 |  | 0.7852 |
| 135 |  | 0.8377 |  | 0.7916 |
| 136 |  | 0.8434 |  | 0.8013 |
| 137 |  | 0.8512 |  | 0.8023 |
| 138 |  | 0.8597 |  | 0.8128 |
| 139 |  | 0.8649 |  | 0.8236 |
| 140 |  | 0.8655 |  | 0.8288 |
| 141 |  | 0.8667 |  | 0.8315 |
| 142 |  | 0.8717 |  | 0.8341 |
| 143 |  | 0.8862 |  | 0.8492 |
| 144 |  | 0.9284 |  | 0.8565 |
| 145 |  | 0.7645 |  | 0.8589 |
| 146 |  | 0.8309 |  | 0.8628 |
| 147 |  | 0.8321 |  | 0.8748 |
| 148 |  | 0.8589 |  | 0.8784 |
| 149 |  | 0.8718 |  | 0.7828 |
| 150 |  | 0.8719 |  | 0.7962 |
| 151 |  | 0.8748 |  | 0.8120 |
| 152 |  | 0.7624 |  | 0.8257 |
| 153 |  | 0.8280 |  | 0.8338 |
| 154 |  | 0.8387 |  | 0.8437 |
| 155 |  | 0.8570 |  | 0.8476 |
| 156 |  | 0.8590 |  | 0.8500 |
| 157 |  | 0.8684 |  | 0.8548 |
| 158 |  | 0.8702 |  | 0.8568 |
| 159 |  | 0.8715 |  | 0.8703 |
| 160 |  | 0.8931 |  | 0.8786 |
| 161 |  | 0.8996 |  | 0.7805 |
| 162 |  | 0.9121 |  | 0.7806 |
| 163 |  | 0.8199 |  | 0.7910 |
| 164 |  | 0.8225 |  | 0.7935 |
| 165 |  | 0.8277 |  | 0.8021 |
| 166 |  | 0.8334 |  | 0.8065 |
| 167 |  | 0.8529 |  | 0.8201 |
| 168 |  | 0.8572 |  | 0.8263 |
| 169 |  | 0.8620 |  | 0.8316 |
| 170 |  | 0.8633 |  | 0.8338 |
| 171 |  | 0.8641 |  | 0.8477 |
| 172 |  | 0.8658 |  | 0.8559 |
| 173 |  | 0.8682 |  | 0.8599 |
| 174 |  | 0.8711 |  | 0.8631 |
| 175 |  | 0.9060 |  | 0.8732 |
| 176 |  | 0.7954 |  | 0.8790 |
| 177 |  | 0.8158 |  | 0.8081 |
| 178 |  | 0.8269 |  | 0.7879 |
| 179 |  | 0.8272 |  | 0.8367 |
| 180 |  | 0.8397 |  | 0.7664 |
| 181 |  | 0.8429 |  | 0.7790 |
| 182 |  | 0.8458 |  | 0.8053 |
| 183 |  | 0.8566 |  | 0.8167 |
| 184 |  | 0.8587 |  | 0.8366 |
| 185 |  | 0.8605 |  | 0.8373 |
| 186 |  | 0.8628 |  | 0.8408 |
| 187 |  | 0.8653 |  | 0.8424 |
| 188 |  | 0.8728 |  | 0.8470 |
| 189 |  | 0.8764 |  | 0.8556 |
| 190 |  | 0.8799 |  | 0.7704 |
| 191 |  | 0.8802 |  | 0.7784 |
| 192 |  | 0.8917 |  | 0.8070 |
| 193 |  | 0.8962 |  | 0.8235 |
| 194 |  | 0.6043 |  | 0.8310 |
| 195 |  | 0.7931 |  | 0.8365 |
| 196 |  | 0.8258 |  | 0.8417 |
| 197 |  | 0.8284 |  | 0.8432 |
| 198 |  | 0.8332 |  | 0.8433 |
| 199 |  | 0.8444 |  | 0.8457 |
| 200 |  | 0.8457 |  | 0.8496 |
| 201 |  | 0.8502 |  | 0.8563 |
| 202 |  | 0.8586 |  | 0.8609 |
| 203 |  | 0.8602 |  | 0.8645 |
| 204 |  | 0.8606 |  | 0.8736 |
| 205 |  | 0.8637 |  | 0.8764 |
| 206 |  | 0.8661 |  | 0.7017 |
| 207 |  | 0.8698 |  | 0.7686 |
| 208 |  | 0.8714 |  | 0.7883 |
| 209 |  | 0.8741 |  | 0.7957 |
| 210 |  | 0.8764 |  | 0.7968 |
| 211 |  | 0.8788 |  | 0.8011 |
| 212 |  | 0.8792 |  | 0.8211 |
| 213 |  | 0.8834 |  | 0.8221 |
| 214 |  | 0.8851 |  | 0.8284 |
| 215 |  | 0.8919 |  | 0.8291 |
| 216 |  | 0.8938 |  | 0.8319 |
| 217 |  | 0.9415 |  | 0.8353 |
| 218 |  | 0.7551 |  | 0.8411 |
| 219 |  | 0.8186 |  | 0.8420 |
| 220 |  | 0.8202 |  | 0.8452 |
| 221 |  | 0.8262 |  | 0.8471 |
| 222 |  | 0.8265 |  | 0.8490 |
| 223 |  | 0.8476 |  | 0.8573 |
| 224 |  | 0.8499 |  | 0.8625 |
| 225 |  | 0.8504 |  | 0.8645 |
| 226 |  | 0.8614 |  | 0.8728 |
| 227 |  | 0.8627 |  | 0.8771 |
| 228 |  | 0.8643 |  | 0.8785 |
| 229 |  | 0.8694 |  | 0.8787 |
| 230 |  | 0.8855 |  | 0.9042 |
| 231 |  | 0.9020 |  | 0.9051 |

Note: channel 5 and 3 from cartridge 1, channel 4 from cartridge 2 and channel 2 from cartridge 3.

Table S5. The number of positive and negative partition per replicate on QuantStudio 12k digital PCR

| Vials | Replicates | Negative | Positive | Mean copies/partition | Filled partitions |
| --- | --- | --- | --- | --- | --- |
| Vial-1 | 1 | 13 | 51 | 1.59 | 64 |
|  | 2 | 14 | 50 | 1.52 | 64 |
|  | 3 | 13 | 50 | 1.58 | 63 |
|  | 4 | 14 | 50 | 1.52 | 64 |
|  | 5 | 13 | 50 | 1.58 | 63 |
| Vial-2 | 1 | 14 | 50 | 1.52 | 64 |
|  | 2 | 14 | 50 | 1.52 | 64 |
|  | 3 | 13 | 51 | 1.59 | 64 |
|  | 4 | 14 | 50 | 1.52 | 64 |
|  | 5 | 14 | 50 | 1.52 | 64 |
| Vial-3 | 1 | 13 | 51 | 1.59 | 64 |
|  | 2 | 14 | 50 | 1.52 | 64 |
|  | 3 | 14 | 50 | 1.52 | 64 |
|  | 4 | 14 | 50 | 1.52 | 64 |
|  | 5 | 14 | 50 | 1.52 | 64 |

Table S6. The number of positive and negative partition per replicate on BioMark digital PCR

| Vials | Replicates | Negative | Positive | Mean copies/partition | Filled partitions |
| --- | --- | --- | --- | --- | --- |
| Vial-1 | 1 | 167 | 598 | 1.52 | 765 |
|  | 2 | 166 | 599 | 1.53 | 765 |
|  | 3 | 160 | 605 | 1.56 | 765 |
|  | 4 | 159 | 606 | 1.57 | 765 |
|  | 5 | 163 | 602 | 1.55 | 765 |
| Vial-2 | 1 | 159 | 606 | 1.57 | 765 |
|  | 2 | 161 | 604 | 1.56 | 765 |
|  | 3 | 160 | 605 | 1.56 | 765 |
|  | 4 | 164 | 601 | 1.54 | 765 |
|  | 5 | 158 | 607 | 1.58 | 765 |
| Vial-3 | 1 | 157 | 608 | 1.58 | 765 |
|  | 2 | 163 | 602 | 1.55 | 765 |
|  | 3 | 155 | 610 | 1.60 | 765 |
|  | 4 | 156 | 609 | 1.59 | 765 |
|  | 5 | 162 | 603 | 1.55 | 765 |

Table S7. The number of positive and negative partition per replicate on QX100 digital PCR

| Vials | Replicates | Negative | Positive | Mean copies/droplet | Accepted droplets |
| --- | --- | --- | --- | --- | --- |
| Vial-1 | 1 | 3298 | 11076 | 1.47 | 14374 |
|  | 2 | 3032 | 11006 | 1.53 | 14038 |
|  | 3 | 2987 | 10987 | 1.54 | 13974 |
|  | 4 | 2897 | 10023 | 1.50 | 12920 |
|  | 5 | 3021 | 11098 | 1.54 | 14119 |
| Vial-2 | 1 | 3002 | 11007 | 1.54 | 14009 |
|  | 2 | 2957 | 10577 | 1.52 | 13534 |
|  | 3 | 2887 | 10877 | 1.56 | 13764 |
|  | 4 | 2787 | 10077 | 1.53 | 12864 |
|  | 5 | 2789 | 10784 | 1.58 | 13573 |
| Vial-3 | 1 | 2780 | 10564 | 1.57 | 13344 |
|  | 2 | 2945 | 11341 | 1.58 | 14286 |
|  | 3 | 2884 | 11276 | 1.59 | 14160 |
|  | 4 | 3012 | 11077 | 1.54 | 14089 |
|  | 5 | 2987 | 10977 | 1.54 | 13964 |

Table S8. The number of positive and negative partition per replicate on RainDrop digital PCR

| Vials | Replicates | Negative | Positive | Mean copies/droplet | Accepted droplets |
| --- | --- | --- | --- | --- | --- |
| Vial-1 | 1 | 353432 | 1345000 | 1.57 | 1698432 |
|  | 2 | 361245 | 1298798 | 1.53 | 1660043 |
|  | 3 | 392389 | 1319876 | 1.47 | 1712265 |
|  | 4 | 345654 | 1345903 | 1.59 | 1691557 |
|  | 5 | 398764 | 1345004 | 1.48 | 1743768 |
| Vial-2 | 1 | 381763 | 1344989 | 1.51 | 1726752 |
|  | 2 | 348978 | 1315006 | 1.56 | 1663984 |
|  | 3 | 354435 | 1343548 | 1.57 | 1697983 |
|  | 4 | 378764 | 1319001 | 1.50 | 1697765 |
|  | 5 | 348732 | 1299690 | 1.55 | 1648422 |
| Vial-3 | 1 | 398764 | 1301201 | 1.45 | 1699965 |
|  | 2 | 368768 | 1328011 | 1.53 | 1696779 |
|  | 3 | 398764 | 1289012 | 1.44 | 1687776 |
|  | 4 | 398764 | 1316003 | 1.46 | 1714767 |
|  | 5 | 369732 | 1315014 | 1.52 | 1684746 |


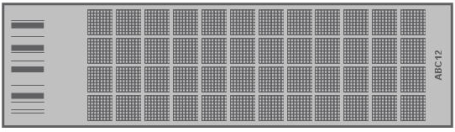


1. Weighing the empty chip
2. Loading the empty chip


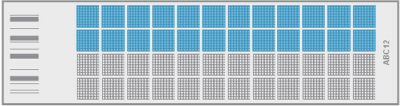

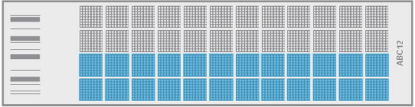

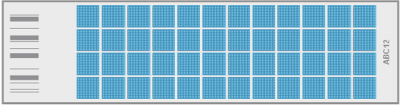


1. Covering and weighing the loaded chip
2. Imaging the loaded chip


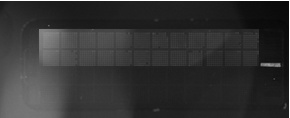

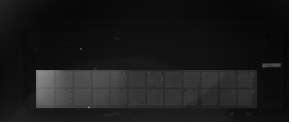

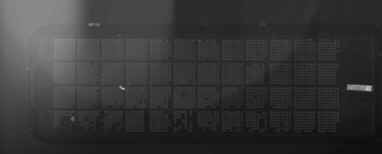


Figure S1. Workflow for measuring the partition volume of the OpenArray® chip by gravimetric analysis.


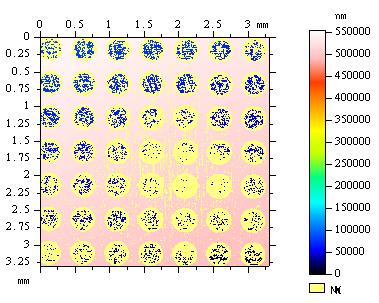

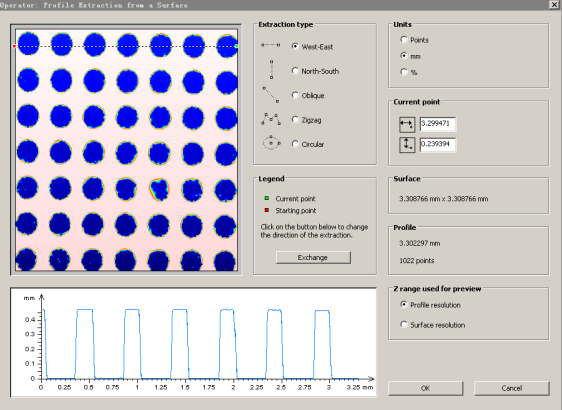


a. Contour map

c. The height measurement of through-hole

b. 3D profile figure

d. The radius measurement of through-hole


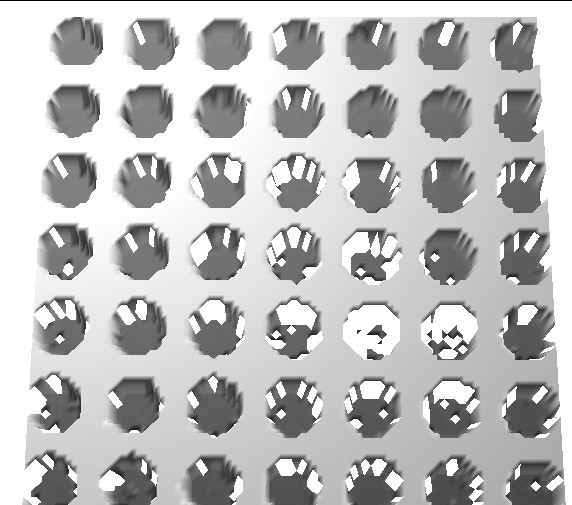

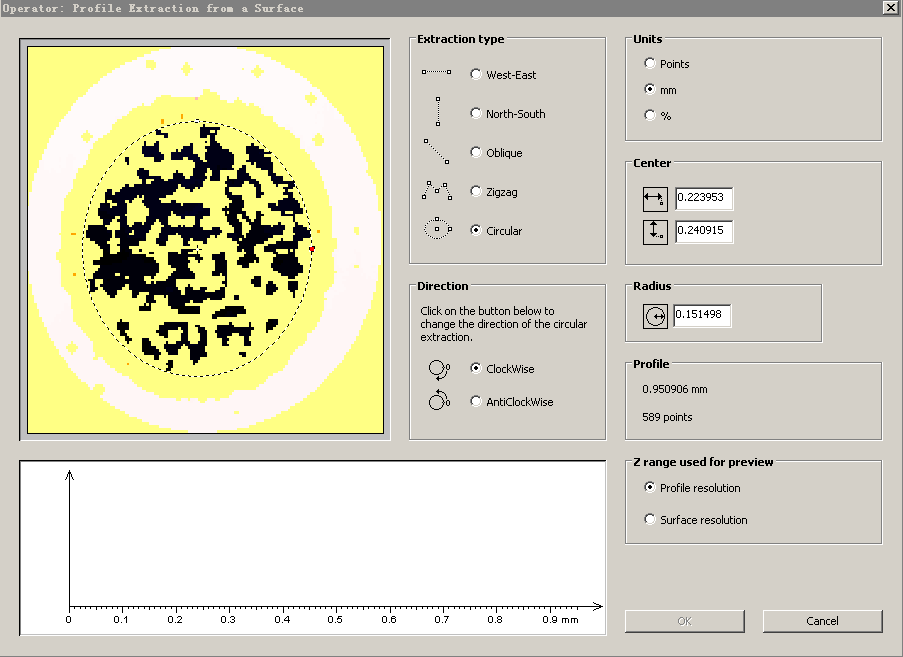


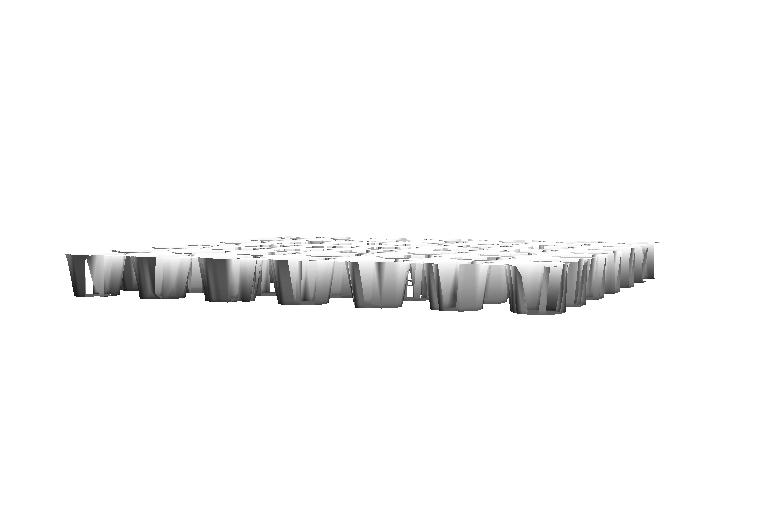


Figure S2. Image and measurement of the through-hole on the OpenArray® chip by Talysurf CCI-Lite Non-contact 3D Profiler, a Coherence Correlation Interferometer (patented by Taylor Hobson). Four repeat measurements were performed by Talysurf CCI fitted with a 5× magnification objective lens in a continuous period of time. (a), contour map, showing elevations and surface configuration by means of pseudo colors; (b), reconstructed 3D profile figure of the through-hole; (c), the height measurement of through-hole when fixing the bottom; (d), the radius measurement of through-hole, a random hole was selected for the radius measurement. The averaged volume with a standard deviation of the four measurements for each hole was (34.18±0.34) nL calculated based on the through-hole was a perfect cylinder.


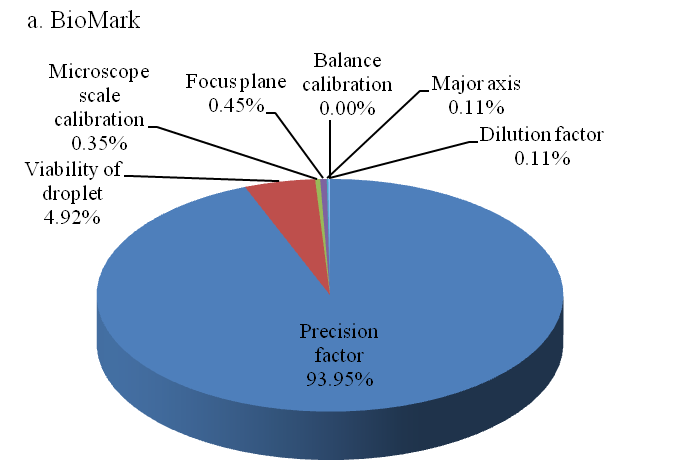


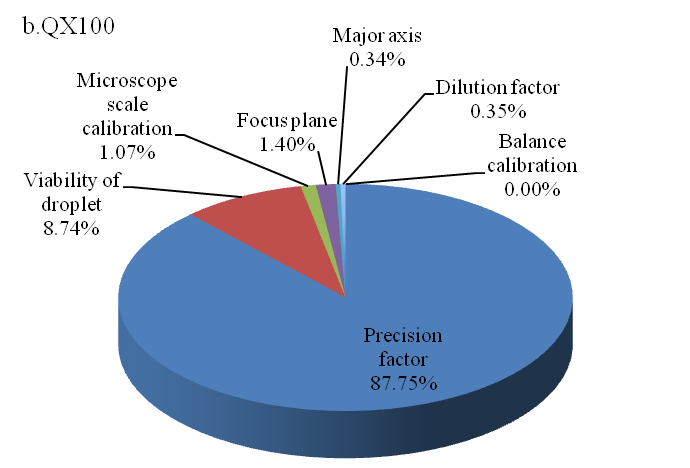


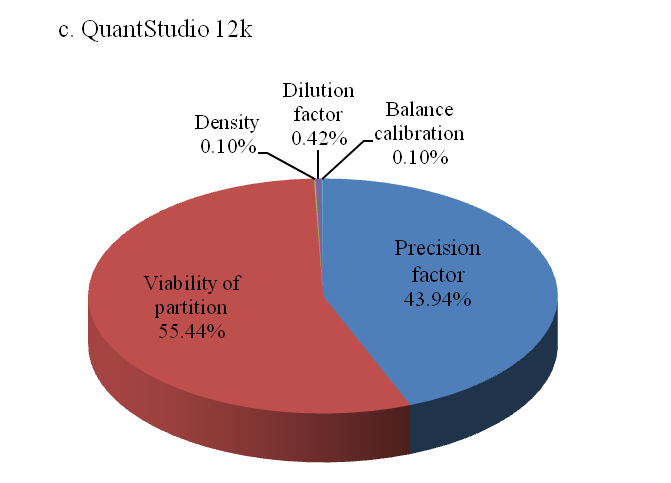


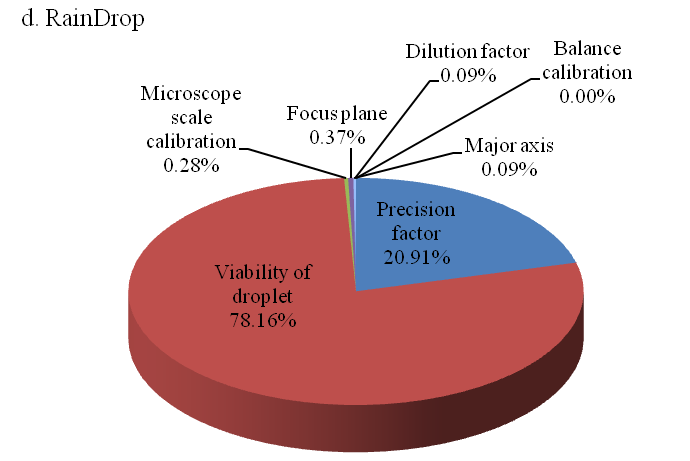


Figure S3. The uncertainty evaluation chart of four digital PCR measurement systems. (a), BioMark, (b), QX100, (c), QuantStudio 12K, and (d), RainDrop.

Figure S4. Stock concentration with the ex
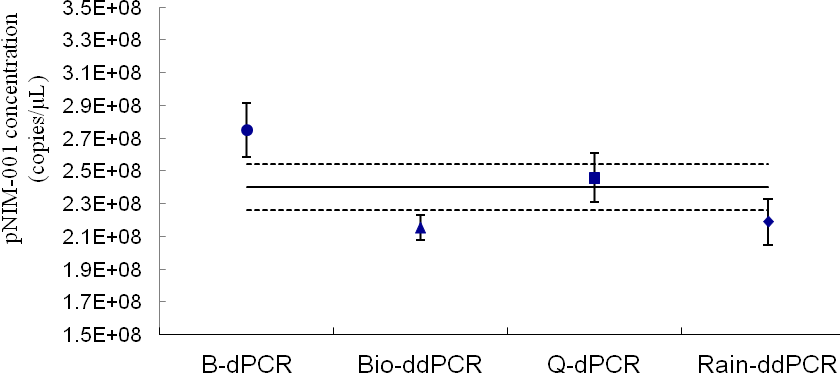
panded uncertainty of the certified plasmid DNA measured by BioMark (B-dPCR), BioRad QX100 droplet digital PCR (Bio-ddPCR), QuantStudio 12K flex digital PCR (Q-dPCR) and RainDrop (Rain-ddPCR), without correction of partition volume of each dPCR. The certified concentration for the plasmid DNA stock (black line) with the expanded uncertainty (dash lines) was provided by isotope dilution mass spectrometry and B-dPCR with partition volume correction.
